# Towards String Support in JayHorn (Competition Contribution)

**DOI:** 10.1007/978-3-030-72013-1_29

**Published:** 2021-02-26

**Authors:** Ali Shamakhi, Hossein Hojjat, Philipp Rümmer

**Affiliations:** 8 Eindhoven University of Technology, Eindhoven, The Netherlands; 9 Aalborg University, Aalborg East, Denmark; 10 University of Tehran, Tehran, Iran; 11 Tehran Institute for Advanced Studies, Tehran, Iran; 12 Uppsala University, Uppsala, Sweden

## Abstract

JayHorn is a Horn clause-based model checker for Java programs that has been competing at SV-COMP since 2019. An ongoing research and implementation effort is to add support for String data-type to JayHorn. Since current Horn solvers do not support strings natively, we consider a representation of (unbounded) strings using algebraic data-types, more precisely as lists. This paper discusses Horn clause encodings of different string operations, and presents preliminary results.

## 1 The JayHorn Approach and Architecture

We start by summarising the approach used in JayHorn, and refer to earlier papers [5,6,7] for more details. JayHorn is a verification tool that encodes sequential Java programs as sets of Constrained Horn Clauses (CHCs) in order to check for possible assertion violations. The main CHC encoding in JayHorn is inspired by refinement types [2] and liquid types [8], and characterises programs in terms of *method contracts, state invariants,* and *instance invariants* of classes [5]. This encoding is over-approximate, and can prove absence of assertion violations. In order to find counterexamples, i.e., prove existence of violations, JayHorn also offers a bounded, under-approximate program encoding.

JayHorn is entirely implemented in Java, and uses the Soot framework [10] to process Java bytecode, and the CHC solver Eldarica [3] to solve Horn clauses.

## 2 Encoding of String Operations

In this paper, we focus on the handling of `Strings` and their operations, a feature of Java that was not previously supported by JayHorn. Since JayHorn verifies programs without imposing bounds on the number of execution steps or the size of input data, our goal is to handle also unbounded strings. Unfortunately, while there has been significant progress in SMT solving for strings, current CHC solvers do not yet support strings natively. We therefore use recursive algebraic data types to model strings, and follow the approach proposed in [4]: strings are represented using lists, with a binary constructor cons and the constant nil.

There are two ways to encode a string using cons and nil. The Left-To-Right (LTR) encoding starts with the leftmost character of the string. For example, "Jay" = cons('J', cons('a', cons('y', nil))). The Right-to-Left (RTL) encoding starts with the rightmost character. Each encoding has its own benefits and drawbacks in modeling various operations, an aspect we evaluate in this paper.

Three different LTR encodings of the concatenation operation are described in [4], and equivalent RTL encodings are easy to define. Moving beyond concatenation, in this paper we show models of some of the more involved operations.

## 2.1 The CompareTo Operation

The `String.compareTo` method in Java returns an integer, which is the difference of the length of strings if one of the strings is a prefix of the other (e.g., `"cat".compareTo("c") == 2`), or the difference of their leftmost same-index different characters otherwise (e.g., `"card".compareTo("cash") == -1`, since their leftmost same-index different characters are 'r' and 's', respectively).

The method is modeled using predicate $P_{rec}(\mathit{left}, \mathit{right}, \mathit{comparison\_result})$ under LTR encoding, which allows us to recursively remove leftmost characters from both strings to reach a state which the *comparison_result* is known.

$$\begin{aligned}
P_{rec}(x, \mathsf{nil}, \mathsf{len}(x)) &\leftarrow \mathit{true}\\
P_{rec}(\mathsf{nil}, y, -\mathsf{len}(y)) &\leftarrow \mathit{true}\\
P_{rec}(x, x, 0) &\leftarrow \mathit{true}\\
P_{rec}(\mathsf{cons}(j, x), \mathsf{cons}(k, y), j - k) &\leftarrow j \neq k\\
P_{rec}(\mathsf{cons}(h, x), \mathsf{cons}(h, y), d) &\leftarrow P_{rec}(x, y, d)
\end{aligned}$$

The predicate under RTL encoding needs an extra argument to keep track of whether the *comparison_result* is based on character difference or not, so the predicate is $P'_{rec}(\mathit{left}, \mathit{right}, \mathit{comparison\_result}, \mathit{char\_diff})$. The clauses use the len function to compute the length of a string, which is a built-in function in Eldarica.

$$\begin{aligned}
P'_{rec}(x, \mathsf{nil}, \mathsf{len}(x), \mathit{false}) &\leftarrow \mathit{true}\\
P'_{rec}(\mathsf{nil}, y, -\mathsf{len}(y), \mathit{false}) &\leftarrow \mathit{true}\\
P'_{rec}(x, x, 0, \mathit{false}) &\leftarrow \mathit{true}\\
P'_{rec}(\mathsf{cons}(h, x), y, d + 1, \mathit{false}) &\leftarrow P'_{rec}(x, y, d, \mathit{false}) \wedge \mathsf{len}(x) \geq \mathsf{len}(y)\\
P'_{rec}(x, \mathsf{cons}(h, y), d - 1, \mathit{false}) &\leftarrow P'_{rec}(x, y, d, \mathit{false}) \wedge \mathsf{len}(x) \leq \mathsf{len}(y)\\
P'_{rec}(\mathsf{cons}(j, x), \mathsf{cons}(k, x), j - k, \mathit{true}) &\leftarrow j \neq k\\
P'_{rec}(\mathsf{cons}(h, x), y, d, \mathit{true}) &\leftarrow P'_{rec}(x, y, d, \mathit{true})\\
P'_{rec}(x, \mathsf{cons}(h, y), d, \mathit{true}) &\leftarrow P'_{rec}(x, y, d, \mathit{true})
\end{aligned}$$

## 2.2 Integer to String conversion

The integer to string conversion relies on extracting digits one by one, which is done using integer arithmetic. Under LTR encoding, during the conversion process, the pre-condition stores the rest of the input after removing the converted digits so far starting from the lowest position. For example, if the number is

$i = \overline{d_{n-1} \cdots d_0}$ and the converted string so far is $s =$ "$d_{k-1} \cdots d_0$", the rest of the number will be $r = \overline{d_{n-1} \cdots d_k}$ which is stored at the pre-condition.

The pre-condition in RTL encoding stores the offset of the next digit that needs to be extracted, since extracting digits from highest place values requires knowing their positions.

### 2.3 StartsWith and EndsWith

The encoding of `String.startsWith` method needs to consider different states of both strings and their relation, which leads to multiple recursive relations.

For example, if $x$ starts with $y$, we can prepend $c$ to both strings under LTR encoding (to get $x'$ and $y'$) and the condition holds on the resulting strings (i.e. $x'$ starts with $y'$). For another example, if $x$ does not start with $y$ and $\mathsf{len}(x) \geq \mathsf{len}(y)$ we can append $c$ to $x$ under RTL encoding (to get $x'$) and the condition holds on the resulting string (i.e. $x'$ does not start with $y$).

$$
\begin{array}{lrcl}
 & S_{rec}(x, \mathsf{nil}, \mathit{true}) & \leftarrow & \mathit{true} \\
 & S_{rec}(x, x, \mathit{true}) & \leftarrow & \mathit{true} \\
 & S_{rec}(\mathsf{nil}, y, \mathit{false}) & \leftarrow & \mathsf{len}(y) > 0 \\
 & S_{rec}(\mathsf{cons}(j, x), \mathsf{cons}(k, y), \mathit{false}) & \leftarrow & S_{rec}(x, y, \mathit{false}) \\
\text{(LTR)} & S_{rec}(\mathsf{cons}(h, x), \mathsf{cons}(h, y), \mathit{true}) & \leftarrow & S_{rec}(x, y, \mathit{true}) \\
\text{(LTR)} & S_{rec}(\mathsf{cons}(j, x), \mathsf{cons}(k, y), \mathit{false}) & \leftarrow & j \neq k \\
\text{(RTL)} & S_{rec}(\mathsf{cons}(h, x), y, \mathit{true}) & \leftarrow & S_{rec}(x, y, \mathit{true}) \\
\text{(RTL)} & S_{rec}(\mathsf{cons}(j, x), \mathsf{cons}(k, x), \mathit{false}) & \leftarrow & j \neq k \\
\text{(RTL)} & S_{rec}(\mathsf{cons}(h, x), y, \mathit{false}) & \leftarrow & S_{rec}(x, y, \mathit{false}) \wedge \mathsf{len}(x) \geq \mathsf{len}(y) \\
\text{(RTL)} & S_{rec}(x, \mathsf{cons}(h, y), \mathit{false}) & \leftarrow & S_{rec}(x, y, \mathit{false})
\end{array}
$$

The RTL encoding of `endsWith` is the same as LTR encoding of `startsWith`, and the LTR encoding of `endsWith` is the same as RTL encoding of `startsWith`.

### 2.4 CharAt

The encoding definition of `String.charAt` relies on the fact that prepending a character to a string under LTR encoding increases indices of all previous characters by one, while appending a character to a string under RTL encoding does not change those indices.

$$
\begin{array}{lrcl}
\text{(LTR)} & \mathit{ChAt}_{rec}(\mathsf{cons}(h, t), 0, h) & \leftarrow & \mathit{true} \\
\text{(LTR)} & \mathit{ChAt}_{rec}(\mathsf{cons}(h, t), i + 1, c) & \leftarrow & \mathit{ChAt}_{rec}(t, i, c) \wedge 0 \leq i < \mathsf{len}(t) \\
\text{(RTL)} & \mathit{ChAt}_{rec}(\mathsf{cons}(h, t), \mathsf{len}(t), h) & \leftarrow & \mathit{true} \\
\text{(RTL)} & \mathit{ChAt}_{rec}(\mathsf{cons}(h, t), i, c) & \leftarrow & \mathit{ChAt}_{rec}(t, i, c) \wedge 0 \leq i < \mathsf{len}(t)
\end{array}
$$

## 3 Performance of the String Encoding

The following table shows the results of JayHorn on the 53 problems in the SV-COMP Java track that involve strings. Many of the programs contain string operations that are not yet handled in JayHorn, but the results already make it possible to compare encoding choices. Uniformly, RTL performs better than LTR (probably because appending characters to strings is more common than adding characters in the beginning), and the under-approximating CHC encoding of JayHorn performs better than the over-approximate encoding (probably because over-approximation too often loses information about string contents). The choice between Iterative, Recursive, or Recursive-with-precondition [4] for string concatenation surprisingly had no effect on the results.

| Encoding Choices | Iterative | | | | Recursive | | | | RecursiveWithPrec | | | |
|---|---|---|---|---|---|---|---|---|---|---|---|---|
| | U-Approx | | O-Approx | | U-Approx | | O-Approx | | U-Approx | | O-Approx | |
| | LTR | RTL | LTR | RTL | LTR | RTL | LTR | RTL | LTR | RTL | LTR | RTL |
| # Solved | 4 | 6 | 1 | 3 | 4 | 6 | 1 | 3 | 4 | 6 | 1 | 3 |
| Avg. Time (s) | 81 | 79 | 7.5 | 16 | 79 | 78 | 7.6 | 16 | 77 | 78 | 7.7 | 16 |

In other respects, JayHorn performed similarly in SV-COMP 2021 [1] as in the two previous years. JayHorn gave one incorrect answer, for the problem `UnsatAddition02` and due to the use of unbounded integer arithmetic instead of correct Java machine arithmetic semantics. JayHorn could correctly prove 125 benchmarks safe, and 151 benchmarks unsafe. Changes compared to 2020 include 59 of the 64 `MinePump` benchmarks (by encoding `enums`, see Section 4) and 6 of the 53 string benchmarks that JayHorn solves now.

The biggest factor influencing the performance of JayHorn in SV-COMP is still the incomplete model of the Java API in JayHorn, given the large number of API tests among the SV-COMP Java benchmarks. Our work on supporting `Strings`, described in this paper, is one of the efforts to address the situation.

## 4 Tool Setup

The version submitted to SV-COMP 2021 is JayHorn version 0.7.5-strings,[4] which is also available on Zenodo [9]. In the configuration used in the competition,[5] JayHorn only applies the Horn solver Eldarica. The Benchexec tool info module is called `jayhorn.py` and the benchmark definition file `jayhorn.xml`. JayHorn competes in the Java category.

Since JayHorn only has incomplete support for Java `enums`, in this year we added a small source transformation tool[6] to JayHorn that has the purpose of replacing `enums` with simple integer variables. The script used in the competition applies the transformation tool to the benchmark source code prior to compilation to bytecode.

4 https://github.com/jayhorn/jayhorn/releases/tag/v0.7.5-strings

5 Java options: `-Xss40000k -Xmx12g`
JayHorn options: `-inline-size 50 -conservative -specs -string-encoding recursiveWithPrec -string-direction rtl`

6 https://github.com/jayhorn/jayhorn/tree/devel/enum-eliminator

## 5 Software Project and Contributors

JayHorn was initially developed by Temesghen Kahsai, Philipp Rümmer, and Martin Schäf, with contributions by Daniel Dietsch, Rody Kersten, Huascar Sanchez, and Valentin Wüstholz [6,7]. Further development of the tool is at the moment mainly carried out by the authors of this paper. JayHorn is open source, and distributed under MIT license on https://github.com/jayhorn/jayhorn.

*Acknowledgements.* The work on JayHorn has been supported by the Swedish Research Council (VR) under grant 2018-04727, by the Swedish Foundation for Strategic Research (SSF) under the project WebSec (Ref. RIT17-0011), and by grants from Microsoft and Amazon Web Services.
